# Supplementary material for: Insecticides, more than herbicides, land use, and climate, are associated with declines in butterfly species richness and abundance in the American Midwest
Source: PLoS One. 2024 Jun 20;19(6):e0304319. doi: 10.1371/journal.pone.0304319 (PMC11189219; doi:10.1371/journal.pone.0304319)
Supplement: S1 File — (DOCX) [file pone.0304319.s001.docx]

**Supporting Information for Van Deynze et al. “Pesticides, more than herbicides, land use, and climate, are associated with declines in butterfly species richness and abundance in the American Midwest”**

**Supplementary Tables**

Table S1: Tally of counties by number of years in the panel

| **Years in panel** | **N, counties** |
| --- | --- |
| 1 | 18 |
| 2 | 6 |
| 3 | 6 |
| 4 | 6 |
| 5 | 3 |
| 6 | 3 |
| 7 | 1 |
| 8 | 1 |
| 9 | 3 |
| 10 | 4 |
| 11 | 4 |
| 12 | 2 |
| 13 | 2 |
| 14 | 2 |
| 16 | 6 |
| 17 | 12 |

Table S2: Assignment of active ingredients to pesticide groups

| **Pest Control Group** | **Active Ingredients** |
| --- | --- |
| Bt Traited Seed | N/A |
| Neonicotinoids | Thiamethoxam, clothianidin, imidacloprid |
| Organophosphates | Azinphos-methyl, chlorpyrifos, dicrotophos, diazinon, dimethoate, ethoprophos, fonofos, malathion, methyl parathion, methamidophos, mevinphos, parathion, phorate, phosmet, profenofos, tebupirimphos, terbufos |
| Pyrethroids | Bifenthrin, permethrin, cypermethrin, cyfluthrin, deltamethrin, fenvalerate, cyfluthrin, cyhalothrin-gamma, cyhalothrin-lambda, pyrethrins, esfenvalerate, fenpropathrin, tefluthrin, tralomethrin |
| Non-Glyphosate | All other herbicide active ingredients |
| Glyphosate | Glyphosate |

Table S2: Assignment of NLCD classifications to land cover groups

| Land Cover Group | NLCD Classification |
| --- | --- |
| Cropland | Cultivated crops |
| Forest | Deciduous forest, evergreen forest, mixed forest |
| Urban | Developed - open space, developed - low intensity, developed - medium intensity, developed - high intensity |
| Other (baseline) | Barren land (rock/sand/clay), grassland/herbaceous, pasture/hay, shrub/scrub, open water, perennial ice/snow, woody wetlands, emergent herbaceous wetlands |

| Table S3: Generalized linear model (negative binomial) regression summaries. Full panel. | | | |
| --- | --- | --- | --- |
| Term | All species | Distinct species | Danaus plexippus |
| Intercept | -2.3 (9.05) | -2.12 (9.21) | -18.4 (12.5) |
| Glyphosate area-treatments | 0.285 (0.397) | 1.16*** (0.297) | 0.339 (0.616) |
| Non-glyphosate area-treatments | 0.205 (0.2) | -0.278* (0.145) | 0.033 (0.271) |
| Pyrethroid area-treatments | -0.177 (0.721) | -0.825 (0.627) | -1.16 (1.25) |
| Organophosphate area-treatments | -0.752 (1.22) | 0.757 (0.857) | -0.834 (1.67) |
| Neonicotinoid area-treatments | -0.29 (0.628) | -0.714* (0.411) | -2.51*** (0.871) |
| Bt seed area-treatments | -0.133 (0.728) | -0.257 (0.544) | 1.4 (1.23) |
| Forest land cover | -11.6 (9.79) | 12.3 (9.29) | -18.3 (14.7) |
| Urban land cover | 3.13 (6.44) | -3.93 (6.82) | 7.33 (9.4) |
| Cropland land cover | 4.47 (10.6) | -1.53 (10.7) | 21.9 (14.5) |
| GDD (Early) | -0.00127* (0.000772) | 0.000718 (0.000639) | 0.00124 (0.00127) |
| GDD (Mid) | -0.000394 (0.000487) | 0.000454 (0.00043) | -0.00286*** (0.000897) |
| GDD (Late) | -0.000345 (0.000551) | 0.000729* (0.000432) | -0.000297 (0.00098) |
| Precipitation (Early) | 0.000042 (0.000353) | 0.0000147 (0.000303) | 0.00012 (0.000617) |
| Precipitation (Mid) | -0.00116*** (0.000333) | 0.0000815 (0.000305) | 0.00111** (0.000557) |
| Precipitation (Late) | 0.0000491 (0.00025) | 0.000349 (0.000218) | 0.0000942 (0.000401) |
| Fixed effects | County, Year | County, Year | County, Year |
| Adj. McFadden’s Pseudo R2 | 0.684 | 0.823 | 0.767 |
| Dispersion (theta) | 11.55  (0.672) | 19.87  (1.509) | 4.81  (0.348) |
| AIC | 9,274.6 | 5,593.7 | 5,463.5 |
| BIC | 9,778.2 | 6,097.3 | 5,948.5 |
| N | 637 | 637 | 632 |
| * p < 0.1, ** p < 0.05, *** p < 0.01; Heteroskedasticity-consistent standard errors in parentheses (HC1). | | | |

| Table S4: Generalized linear model (negative binomial) regression summaries. Estimated on panel limited to counties with three or more years of data. | | | |
| --- | --- | --- | --- |
| Term | All species | Distinct species | Danaus plexippus |
| Intercept | -33.1***  (11.6) | 0.0857  (8.65) | -4.2  (8.24) |
| Glyphosate area-treatments | 0.14  (0.287) | 0.485***  (0.141) | 0.0502  (0.167) |
| Non-glyphosate area-treatments | -0.00298  (0.11) | -0.0877  (0.0566) | 0.0471  (0.075) |
| Pyrethroid area-treatments | 0.297  (0.535) | -0.13  (0.27) | -0.171  (0.266) |
| Organophosphate area-treatments | -2.23***  (0.781) | -0.682*  (0.407) | -0.652  (0.421) |
| Neonicotinoid area-treatments | -0.866  (0.556) | -0.187  (0.295) | 0.144  (0.398) |
| Bt seed area-treatments | 0.924*  (0.529) | -0.288  (0.272) | 0.214  (0.319) |
| Forest land cover | -30.5**  (14.4) | 10.4  (8.73) | -17.8*  (10.2) |
| Urban land cover | 19.9**  (8.9) | -5.18  (6.42) | 4.85  (5.86) |
| Cropland land cover | 40.4***  (13.3) | -3.6  (9.89) | 7.44  (9.37) |
| GDD (Early) | 0.00139  (0.00123) | 0.000559  (0.000689) | -0.00126*  (0.000746) |
| GDD (Mid) | -0.0029***  (0.000876) | 0.0004  (0.00045) | -0.000389  (0.000479) |
| GDD (Late) | -0.000144  (0.000993) | 0.000602  (0.000449) | -0.000302  (0.000547) |
| Precipitation (Early) | 0.000184  (0.000622) | -0.0000287  (0.000305) | -0.0000393  (0.000355) |
| Precipitation (Mid) | 0.00108**  (0.000548) | -0.0000428  (0.000291) | -0.00114***  (0.000315) |
| Precipitation (Late) | 0.0000783  (0.000396) | 0.000322  (0.000212) | -0.0000183  (0.000237) |
| Fixed effects | County, Year | County, Year | County, Year |
| Adj. McFadden’s Pseudo R2 | 0.757 | 0.818 | 0.660 |
| Dispersion (theta) | 11.175  (0.665) | 19.05  (1.46) | 4.74  (0.349) |
| AIC | 5285.6 | 5345.4 | 8898.9 |
| BIC | 5677.9 | 5737.7 | 9291.3 |
| N | 607 | 607 | 607 |
| * p < 0.1, ** p < 0.05, *** p < 0.01; Heteroskedasticity-consistent standard errors in parentheses (HC1). | | | |

| Table S5: Generalized mixed linear model (negative binomial) regression summaries. Full panel. | | | |
| --- | --- | --- | --- |
| Term | All species | Distinct species | Danaus plexippus |
| Intercept | -1.55**  (0.645) | -2.35***  (0.562) | -3.2***  (1.13) |
| Glyphosate area-treatments | 0.0828  (0.146) | 0.264**  (0.124) | 0.096  (0.268) |
| Non-glyphosate area-treatments | 0.0097  (0.0632) | -0.00461  (0.0536) | 0.0445  (0.116) |
| Pyrethroid area-treatments | -0.115  (0.258) | -0.347  (0.226) | 0.29  (0.468) |
| Organophosphate area-treatments | -0.753*  (0.402) | -0.686**  (0.337) | -2.08***  (0.737) |
| Neonicotinoid area-treatments | -0.376*  (0.224) | -0.607***  (0.17) | -0.908*  (0.515) |
| Bt seed area-treatments | 0.228  (0.278) | -0.0621  (0.242) | 1.65***  (0.496) |
| Forest land cover | 2.05**  (0.89) | -0.986  (0.784) | -1.36  (1.49) |
| Urban land cover | 1.4*  (0.715) | -3.02***  (0.631) | 0.687  (1.2) |
| Cropland land cover | 1.27*  (0.676) | -0.949  (0.596) | 1.22  (1.14) |
| GDD (Early) | -0.00071  (0.000604) | 0.000262  (0.000484) | 0.00189  (0.00117) |
| GDD (Mid) | -0.0000325  (0.000382) | 0.000552*  (0.000294) | -0.00267***  (0.000775) |
| GDD (Late) | 0.000547  (0.000377) | 0.000246  (0.000286) | 0.000889  (0.000754) |
| Precipitation (Early) | -0.000273  (0.000335) | 0.000162  (0.000281) | 0.0000628  (0.000609) |
| Precipitation (Mid) | -0.000956***  (0.000278) | 0.0000751  (0.000227) | 0.00136***  (0.00051) |
| Precipitation (Late) | -0.0000305  (0.000229) | 0.000161  (0.000196) | 0.000281  (0.000404) |
| Random effects | County, Year | County, Year | County, Year |
| AIC | 9444 | 5741 | 5647 |
| BIC | 9529 | 5825 | 5732 |
| N | 637 | 637 | 632 |
| * p < 0.1, ** p < 0.05, *** p < 0.01 | | | |

Table S6: Pest control coefficient estimates with other pest control covariates omitted. Each row represents a separate model estimate.

| Total Abundance | |  |  |  |
| --- | --- | --- | --- | --- |
|  | Estimate | Std. Error | z value | Pr(>\|z\|) |
| Glyphosate | 0.146 | 0.145 | 1.013 | 0.311 |
| Non glyphosate | -0.026 | 0.067 | -0.392 | 0.695 |
| Pyrethroids | -0.378 | 0.233 | -1.622 | 0.105 |
| Organophosphates | -0.796 | 0.343 | -2.320 | 0.020 |
| Neonicotinoids | 0.406 | 0.329 | 1.237 | 0.216 |
| Bt traited seed | 0.191 | 0.282 | 0.679 | 0.497 |
|  |  |  |  |  |
| Species Richness | |  |  |  |
|  | Estimate | Std. Error | z value | Pr(>\|z\|) |
| Glyphosate | 0.511 | 0.131 | 3.900 | 0.00009613 |
| Non glyphosate | -0.162 | 0.053 | -3.052 | 0.0022715 |
| Pyrethroids | -0.568 | 0.231 | -2.463 | 0.0137904 |
| Organophosphates | -1.030 | 0.337 | -3.055 | 0.0022537 |
| Neonicotinoids | 0.256 | 0.261 | 0.980 | 0.327145 |
| Bt traited seed | 0.047 | 0.257 | 0.181 | 0.856477 |
|  |  |  |  |  |
| Monarch | |  |  |  |
|  | Estimate | Std. Error | z value | Pr(>\|z\|) |
| Glyphosate | 0.295 | 0.255 | 1.156 | 0.24755 |
| Non glyphosate | -0.095 | 0.107 | -0.892 | 0.372277 |
| Pyrethroids | -0.201 | 0.480 | -0.417 | 0.67641 |
| Organophosphates | -1.590 | 0.623 | -2.555 | 0.0106212 |
| Neonicotinoids | 0.076 | 0.493 | 0.154 | 0.877357 |
| Bt traited seed | 0.783 | 0.465 | 1.682 | 0.0924891 |

**Supplementary Figures**


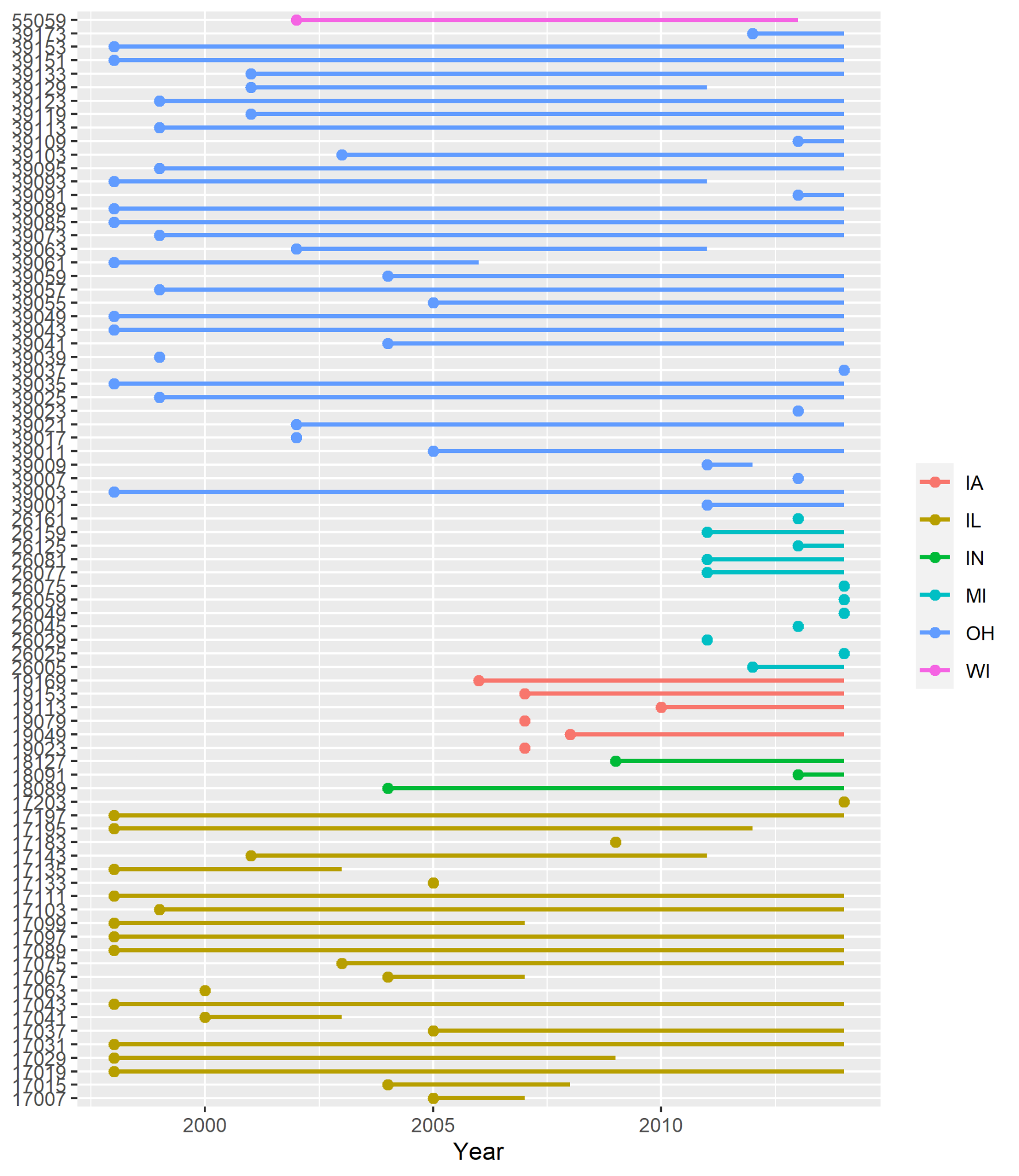


**Fig. S1. Years each county contributes to the panel.** Bold dots indicate first year data is contributed. The vertical axis shows the county’s FIPS code.


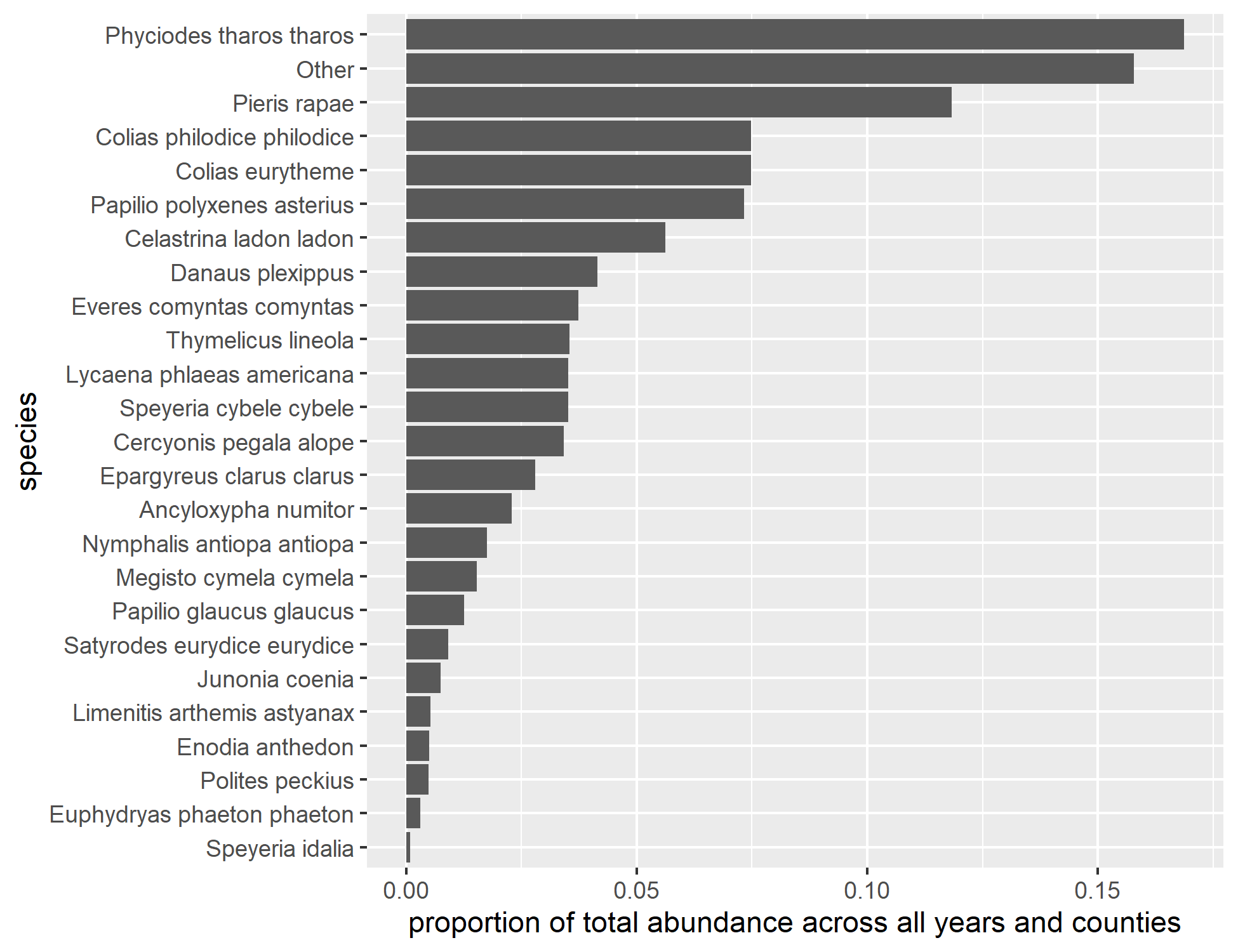


**Fig. S2. Proportion of total abundance across all years and counties by species.**


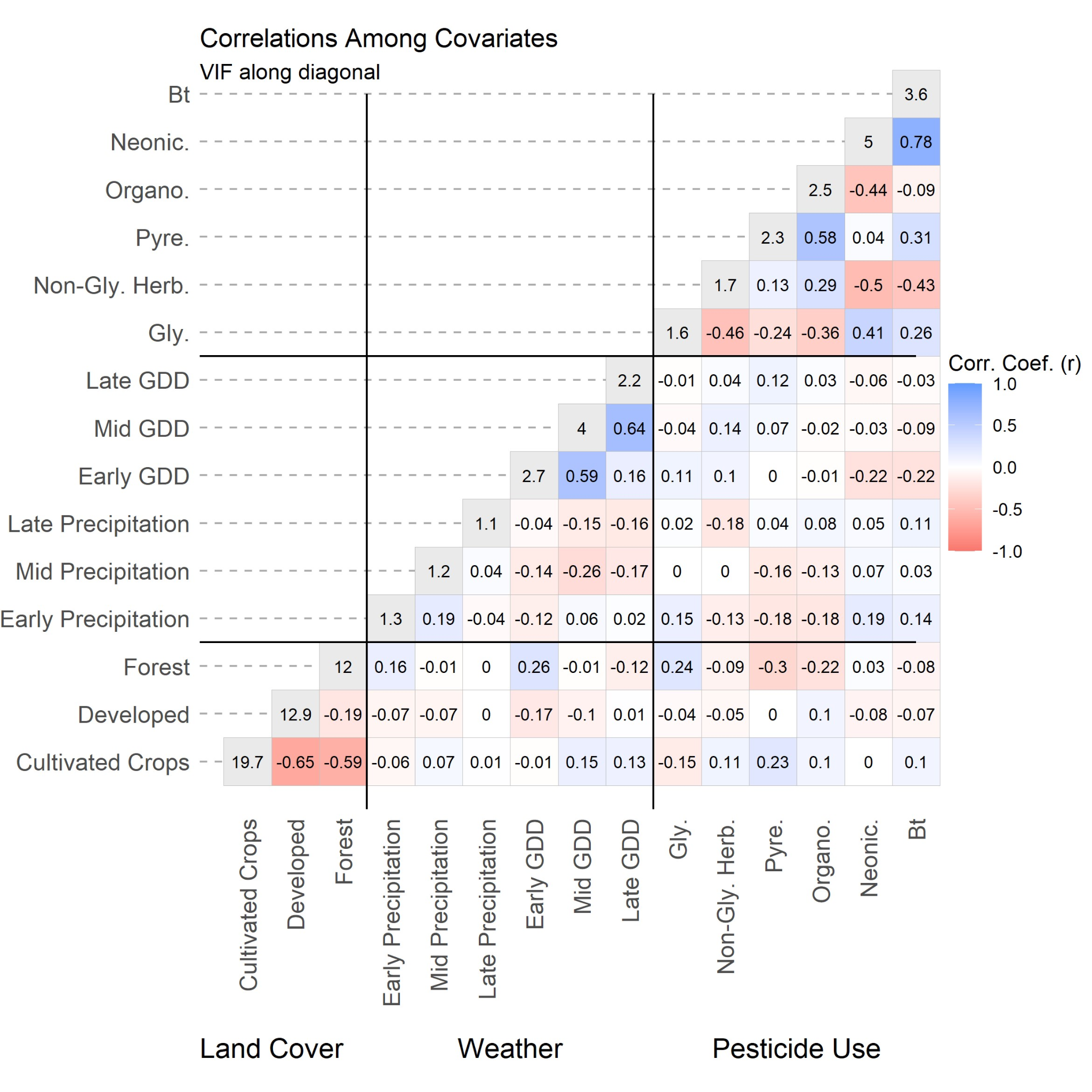
**Figure S3. Pairwise Pearson’s correlation coefficients and Variance Inflation Factors for explanatory variables in the full panel.**

**
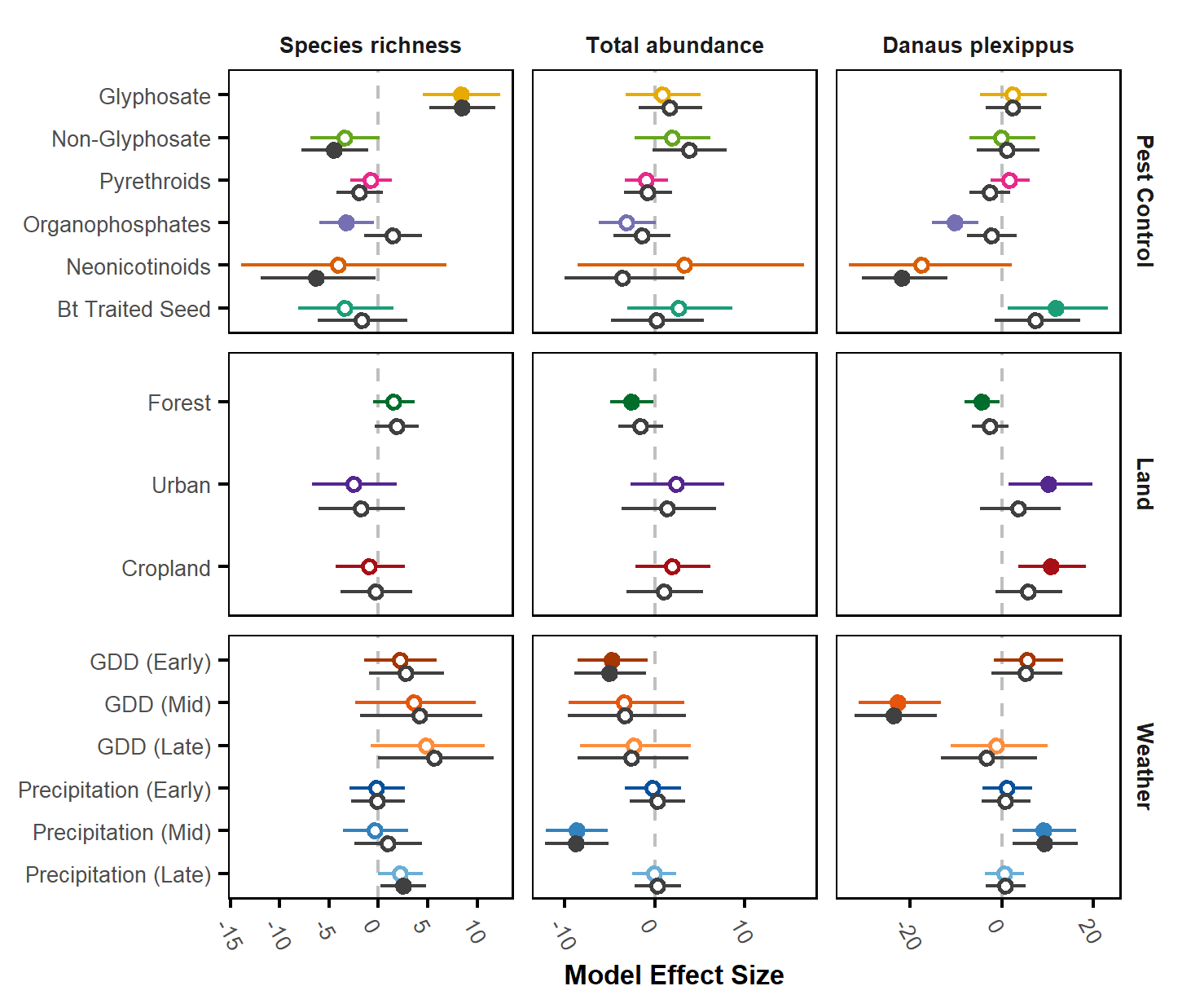
**

**Figure S4. Model effect sizes estimated with panel restricted to counties contributing data three or more years.** Grey points show estimates with full panel for comparison.


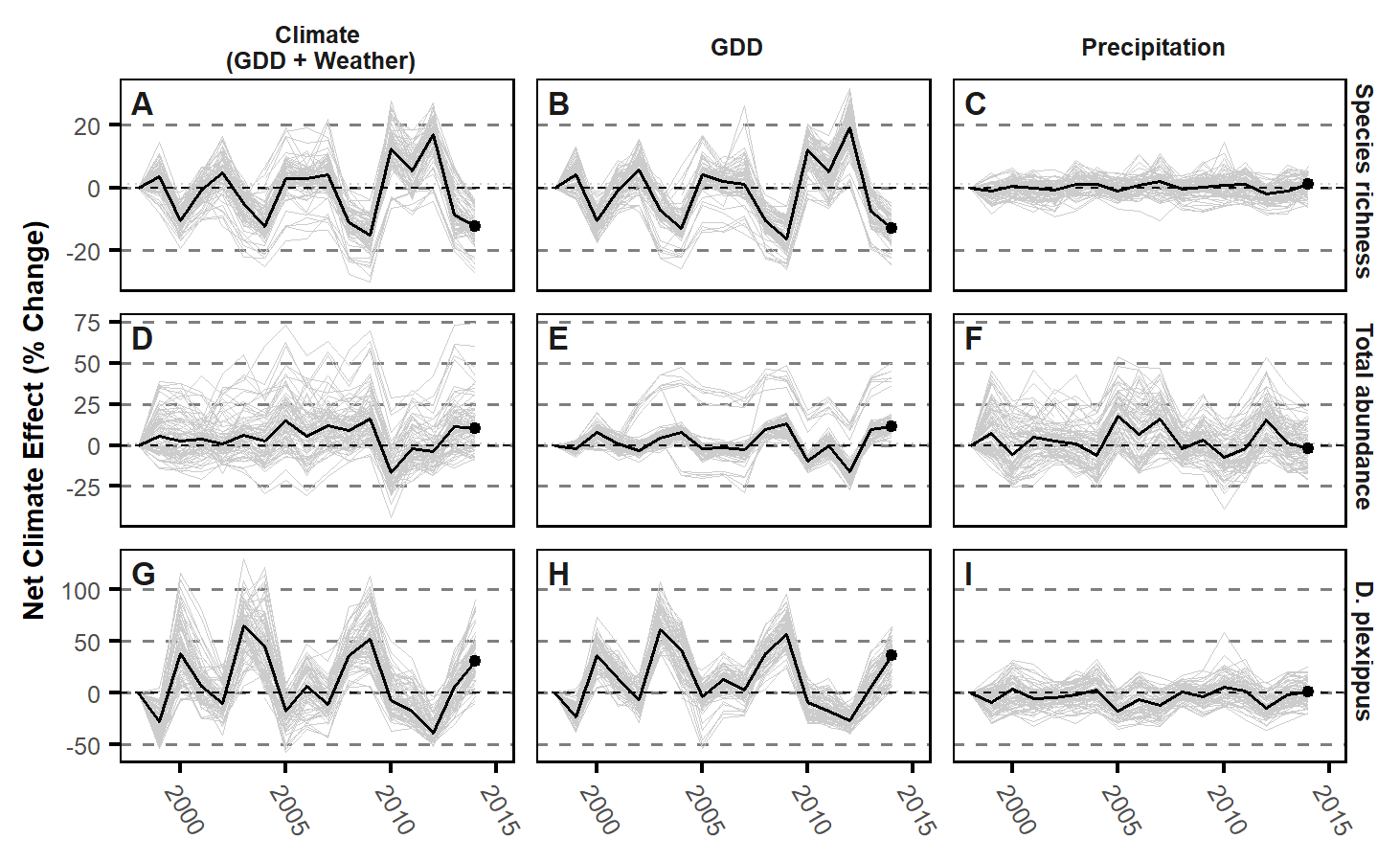


**Fig. S5. Net effect of variation in climate over time.** Net climate effect is the percent change between predicted incidence rates under observed climate variable levels and synthetic counties where climate variable levels are fixed at 1998 levels. County-level net climate effects over time are shown in gray lines and annual median net climate effect in black lines; columns show effects computed holding both GDD and precipitation, GDD alone, and precipitation variables at 1998 levels. Results are presented for species richness (A-C), total abundance (D-F), and *Danus plexippus* (monarch) abundance (G-I).
