# Supplementary material for: Insecticides, more than herbicides, land use, and climate, are associated with declines in butterfly species richness and abundance in the American Midwest
Source: PLoS One. 2024 Jun 20;19(6):e0304319. doi: 10.1371/journal.pone.0304319 (PMC11189219; doi:10.1371/journal.pone.0304319)
Supplement: Attachment — Submitted filename: Response to Reviewers Van Deynze et al revision.docx [file pone.0304319.s002.docx]

**Response to reviews**

(our responses are in blue font)

Additional Editor Comments:

As you may have anticipated, the approach and results presented in this manuscript are polarizing, and led to my expansion of the reviewer number to ensure a fair and thorough review. As you'll see, the reviews range from outright hostility to complete support. I would encourage consideration of criticisms in each of the reviews providing detailed commentary as they get at similar challenges for the reader and the question of whether the conclusions are indeed supported by the data,

There is some question about the methods and exactly how some of the time series contribute to the analysis, especially when there are, for example, only two points in the time series. It would seem that a county with only two surveys would need to be excluded. As it stands, the models are not convincing the reviewers, and will need further explanation (first) and then the question may still remain if they show what they are characterized as showing.

As we discuss below in our responses to reviewers, we have conducted extensive new analyses to address this and other comments about analyses. As to this comment, our original analysis is appropriate, and we maintain that, given the way our model was designed, it is appropriate and strengthens the analysis by leveraging all available data. However, we have conducted an additional analysis that removes data from counties that have three or fewer butterfly surveys.

The reason the full dataset is appropriate is that the GLMs are not an analysis of individual time series. Rather, the independent variables are levels in a given county, and the response variables are butterfly abundance or diversity, in each given year. Thus, it is the full complement of counties in each year that permit us to determine the effects of a level of an independent variable in that year. Take an extreme example: if an extreme drought caused there to be zero butterflies in one year in some counties, and if butterfly abundances in one county were measured only once (when there was extreme drought), that would still give information to inform the model in context of all the other counties for which we have data. We explain the rationale for the full and partial datasets in the Methods (lines 368-371). Inevitably, the reduced dataset had lower statistical power so the results are often not significant but, importantly, the patterns are generally similar (Figure S4).

The overall framing and incorporating the breadth of literature in the field on these topics needs to be revisited, especially if the "blame" for monarch decline is going to be shifted now to neonics the literature on actual effects of neonics on monarchs needs to be evaluated as part of describing the purported mechanism that the models are suggesting. That is, drawing conclusions from the model needs to be supplemented by an engagement of the mechanism by which the purported effect is happening and compared with studies that investigate the direct effect of neonics on monarchs.

The manuscript leaves the impression that glyphosate does not impact monarch population trajectories. You discuss that this is probably because the damage has already been done (first paragraph page 8), but this observation needs to take a much bigger role in structuring the narrative overall if this is to be a blame-shifting paper for monarch populations. The argument in the discussion would need to be that "agricultural practices using glyphosate hammered butterfly habitat in agricultural landscapes and got to a point were so much habitat was gone that additional use had no further impact (the earth was already scorched) and now we are seeing that there is a signal that neonics are having a further impact on monarchs on top of the destruction that occurred already." Assuming this is your argument, of course./

We appreciate that we may not have sufficiently emphasized this apparent disconnect that may arise because of the seeming contrast of our results with past studies. We have added substantial language to explain the historical understanding of causes of monarch decline to put our results in context, both in Box 1 and throughout the discussion (lines 529-572). Importantly, we did not mean for this to come off as “blame shifting” because, as you note, we highlight that we are missing the time frame of the dominant loss of milkweed in the landscape. Indeed, our analyses add the nuance that attributes decline to multiple mechanisms, not just neonicotinoids (as seems to be suggested by our results). We also provide historical perspective on pesticide use that explains why, over time, different drivers may be more important (see Box 1). We address these important issues about the monarch in the revised text and in our responses below.

As a structural matter, please separate out results and discussion. The ms reads like it started its life at *Science* or *Nature*. For PLoS ONE we aren't dealing with those length and style constraints. Should you prepare a revision, it would be appropriate expand the introduction and include a background literature review to address the holes identified by reviewer 1, go into more detail on the methods, report the results separately and without commentary, and then provide a discussion and conclusions that meets PLoS ONE standards ("[A]uthors should avoid overstating their conclusions. Authors may discuss possible implications for their results as long as these are clearly identified as hypotheses instead of conclusions.").

As noted above, we have restructured the revision, including moving content from the supplements to the main text, to more closely adhere to the PLoSONE format.

I am requesting a major revision of the manuscript, but please note that does not guarantee that it will be accepted following revision. An engagement of the comments of each of the reviewers is requested, especially reviewers 2 and 4 relevant to the methods. Reviewer 1 was convinced of the fatal weakness of the manuscript and although pointed out areas where additional context and background information is necessary, did not deeply engage the methods. The "big picture" critiques from reviewer 1 do need to be substantively addressed, however.

We address every comment of reviewer 1 in our response. This review is, in our experience, unique: we have never read a reviewer that is filled with such personal attacks while being almost entirely devoid of any critique of the data or analysis. We address every scientific criticism with a response, including with details of where we addressed a comment with a revision in the manuscript. We address other criticisms as well; coauthor Ries does not want to leave the personal and deeply insulting accusations unanswered. 

**Comments to the Author**

Reviewer #1: This manuscript describes an analysis of multiple butterfly monitoring programs in the American Midwest, over a number of years, and attempted to ascertain if any anthropogenic factors such as pesticides, herbicides, landuse, or climatic variables, influence either the diversity of butterfly species or their abundance. They evaluated all monitored species, but highlighted the effects on monarchs, because (I think) of their popularity, and because of the ongoing narrative about their population trends. There were a number of significant trends with some the variables tested, though the authors highlighted only one of these, which was a negative effect of neonicitinoid pesticides on the species richness, and also on the abundance of monarchs.

I have to say that I was initially very intrigued by the idea of this study, but after reading this paper, I am rather shocked at so many instances where the authors appeared to go out of their way to either mischaracterize or purposely ignore the latest science around butterfly and insect populations here in North America, plus some recent research on monarch butterflies (including their response to neonicitinoids), and even some prior work by one of these labs. I find this very troubling, and it does not speak well of the leadership of the senior authors here. Further, the results of these analyses here seem to be counter to much of the established science just mentioned, which calls the data or analyses into question. If this paper is to go forward (either in this journal or after rejection, to another) then the authors need to do a gut-check, and think hard about how this study will affect their reputations.

**Authors response:** As noted above, we address every specific comment of reviewer 1 below, but here we particularly object to the offensive final statement of this paragraph, which reads as a personal attack and, crucially, is not at all supported by any criticism of our data or analyses.

Specific points

There is so much to point out about this paper that it is hard to know where to start. But for now, let me point out the parts/statements that are mischaracterized. First, the introduction begins with a loaded statement that all insects are declining, which is not entirely true, at least in North America. Indeed, much of this paper is built around this story of insects (butterflies) being in trouble. There have certainly been analyses of long-term data that shows declines of some insects, but then increases in others (Van Klink et al 2020). There has been work showing declines of butterflies in the west (Forister et al 2021) but then a more recent study (Crossley et al 2021) showed this decline was specific to the west, and the butterflies in the east were improving. Another of Forister’s papers showed that some butterfly species are “winning” in the Anthropocene. I note that these studies showing good news were distinctly missing from the text of this paper. Really, the situation is not a straightforward “decline of all insects”, at least in North America.

**Author response:**  We see that in the limited space of the original abstract, our statement that “insects are in decline globally” could be read as “all” species of insects, but we were instead referring to total abundance. In our revised abstract, we have clarified that we are referring to total abundance.  However, in the ***very first*** sentences of our original introduction, we wrote that: *“Globally, declines of abundance and biomass for many insect groups have been reported at rates of 2-4% per year, rates that compound to as much as 30-50% of total abundance loss, depending on study duration.”*  Further, in lines 20-21 on page 2 of our original submission, we wrote: *“... taxon-wide studies for insects show species respond variably, depending on their thermal niche requirements, producing climate “winners” and “losers”.”* Therefore, It is difficult for us to grapple with this accusation. We maintain that our original language had appropriate balance; however, we see that if readers go into the main text of the paper with that misconception, then that will color the way they read the entire paper. Thus, in addition to clarifying our statement in the first sentence of the abstract, and retaining the statements above, we also emphasize this point and add more details in the introduction. We report on papers (lines 39-41) that do not show declines, but also point out that these studies are outliers in the larger literature. For example, while van Klink, et al. (2020) does show an increase in the abundance of aquatic insects, there is an overall decrease in numbers of terrestrial insects, which is much more relevant to our study of butterflies.

Along the same vein, the paper has much language devoted to describing the monarch butterfly as being in trouble, which is also a mischaracterization, based on the latest research (which was also left out here). Some of this research even was authored by one of the authors here. Ries et al 2015 described an analysis of long-term monitoring data from another program, which showed no decline of monarch abundance. Similarly, Inamine et al 2016 described a similar analysis of even more data.

Our abstract echoes reviewer 1’s comment, as it reads: “*This included the abundance of the migratory monarch (Danaus plexippus),* ***whose decline is the focus of intensive debate and public concern***.” As we are consistent with reviewer 1’s sentiment, but contrary to reviewer 1’s comment that we describe “monarchs being in trouble,” we cannot address this comment in any depth in our manuscript. The monarch literature is vast, so much so that it cannot be summarized in this paper. One of us just published a comprehensive summary of population trajectory and driver studies (Shirey and Ries 2023), and that paper’s 8000 words address just one branch of the monarch literature. Importantly, this review shows that the bulk of evidence in the most rigorous studies shows strong population declines in the dominant breeding grounds of both east and west populations. In our resubmission, we cannot address most of reviewer 1’s points because they are outside the scope of this study. However, we did expand on our language, adding 3 paragraphs to emphasize more about the history of monarch studies, so we can more clearly  provide readers with more context about monarch biology (lines 529-572).

To add detail to reviewer 1’s reference to “the author’s own” paper: Ries et al.(2015) and, similarly, Inamine et al., (2016) showed no decline in monarch abundance indices on the summer grounds using NABA and IL data. Thus, the papers that reviewer 1 cites suggested a “disconnect” between the summer (no trend) and winter (declining trends) data (Ries et al., 2015; Inamine et al., 2016). However, in both studies, declines in Mexico were clear and subsequent studies have also shown declines during summer (Zylstra et al., 2021). Indeed, all studies of the overwinter colonies published since 2012 have shown declines both in the eastern and western populations (Shirey and Ries, 2023). Because the winter aggregations provide a snapshot when the entire population is aggregated in one small region, monitoring of the winter colonies is considered the best stage at which to examine the trajectory of the population size over time (regardless of variability during different stages of the migratory cycle).

Crossley et al. 2022 had probably the most comprehensive analysis of monarch data to date, which again, showed no long term decline in the number of summertime monarchs across the continent.

Here, we address what could be construed as a disconnect between the results of a recent publication of monarch population trajectories in summer and winter ranges (especially Zylstra et al., 2021) with the results of Crossley et al. (2022). That study used unweighted averages of population trajectories across the US no matter how big or small the regional monarch populations are, and we find that to be a fatal flaw in their conclusion of no overall decline. This issue was treated in Shirey and Ries 2023 and we quote our summary from Shirley and Ries (2023; see also figure 2 in Crossley, et al. (2022) in support of our interpretation):

*“This is the most comprehensive study showing that across the entire North American range of the monarch, they do not appear to be declining. However, we note that if the spatial patterns of declines, increases, and areas with no apparent trends are examined in their results, there does appear to be a trend towards declines in the Midwest and Northeast, which encompass the main recruitment grounds of the overwinter population in Mexico. Thus, we feel that, despite the interpretation of the authors, our opinion is that this paper actually presents evidence that corroborates our conclusion of monarch declines, at least for the Eastern population.”*

Then, the latest study of the monarch genome (Boyle et al 2023) describes how there has been no long-term decline in the effective population size in the last 75 years. Further, it now looks like the population size now is essentially larger than it ever was prior to human clearing of the prairies and eastern forests 200 years ago.

This comment is beyond the scope of our paper that addresses factors causing changes in population size in recent decades. We point to Shirey and Ries (2023) that acknowledges the Boyle, et al. (2022) study that purports to reconstruct genetic signatures going back 13,000 years to the last glacial maximum. They found that the population likely increased after this time and especially 200 years ago, a result that is not surprising given the clearing of the prairies at that time. Their analysis also suggested there was no genomic evidence of a recent decrease in population size. Since the size of the monarch population has fluctuated between billions and millions over the past decades, we do not agree that the evidence of population trajectories based on the concept of effective population sizes provides credible counter-evidence to trends observed in on-the-ground surveys. Nonetheless, this is not the point of our present paper.

So, the story that monarchs are in decline (even though it is popular in the media) is not accurate based on the latest science. I also note that none of the studies I mentioned were brought up in this paper. I find it hard to believe that this was a simple case of carelessness. More likely the authors did not want to include them, which is highly inappropriate.

We repeat the conservative phrasing in our original abstract (“*This included the abundance of the migratory monarch (Danaus plexippus),* ***whose decline is the focus of intensive debate and public concern***.”) and also note that this was treated with detail in the Shirey and Ries (2023) paper. This is important in our response to Reviewer 1, because revisiting the issue in detail is both unnecessary (given the recent review) and outside the scope of this paper. But to add what we think is the most important recent evidence on declines, we note that the eastern monarch population declined to their lowest level in 2013, at a size 97% lower than it was two decades earlier. From that historic low, it bounced around a new low. It is statistically challenging to determine population trajectories in these cases, and this was specifically treated by Thogmartin et al., (2020). Like other insects, monarch population sizes fluctuate widely. Still, the “high” levels in the past decade suggest at least a 70% decline. As noted by Normile (2023): "Researchers generally agree that the number of monarchs wintering in Mexico declined beginning in the 1990s or earlier but stabilized around 2014 at about 55 million individuals." Indeed, last week we learned that current numbers in Mexico barely exceed the 2013 nadir.

The focus here on the Midwest only did not help to boost my view of this paper either, as it can lead to misleading conclusions. As the Crossley et al 2022 study demonstrates, the butterflies in the U.S. are experiencing declines in some regions, increases in others, and are stable in lots more. So, taking a snapshot of one region (even with a boatload of data) is still just one region, and so the conclusions from this analysis would really only be pertinent to here (i.e. probably could not be extrapolated).

Our response above addresses this comment. We focused on the Midwest because it is a center of agricultural activity in the US and also is the location of the highest density of butterfly monitoring systems in the US (Taron and Ries, 2005). We maintain our position that these results are centered in an agricultural center in the US and have implications for other agricultural regions; we disagree that we inappropriately extrapolated our results.

Moreover, even focusing on the monarchs alone in this region is problematic. The authors state that this is appropriate for monarchs since "95% of monarchs that reach the winter colonies originated in the cornbelt" (citing Wassenaar and Hobson 1998). This is an outdated conclusion, and again, a mischaracterization because the proper (more recent) science was not even mentioned. Wassenaar and Hobson only examined one collection of monarchs from 1997 (an exceptional year). A more thorough study was done by Flockhart et al 2017, in which 40 years of samples were examined. Their results highlighted that the Midwest only produces about 37% of the monarchs that reach Mexico, while 63% come from elsewhere. In fact, their results showed that this varies by year – in some years, most monarchs that reach Mexico come from the northeast, and in others it is the far north. Again, a seeming lack of understanding of the science is the problem here, or perhaps a purposeful effort to neglect the important studies.

We appreciate Reviewer 1’s reference to Flockhart, et al. (2017). We agree it is more accurate to say that the Midwest produces proportionally more arrivals in the overwinter grounds than any other region, and we have modified our text accordingly (lines 240-243).

I’m going to jump right into the main conclusions next for the sake of my time, since there is just too much to go through line-by-line. The main conclusion of this analysis is that neonicitinoid pesticides are causing declines to butterflies, and to monarchs in particular.

This is a vast simplification of our conclusions. Indeed, our study shows that neonics are only one of many contributing factors to yearly variation in the spatial distribution of butterflies in the Midwest, and the relative contribution of neonicotinoids varies widely among our butterfly response variables (now shown in Table S3-S5). However, it emerged as one of the clearest correlates compared to environmental factors driving patterns, especially in richness across different county-years (Fig. 3A-C in our original submission).

This is a result that sounds ridiculous, based on the accumulated laboratory research thus far. The effects of neonics on monarch specifically have been assessed multiple times, by multiple labs, and each time, there is little to no effect found, at least when standard, field-relevant doses are used. For some reason, monarch butterflies are resistant to nearly every neonicitinoid chemical tested, likely because of their ability to take up the toxins from milkweeds. In a few cases, declines have been found, but only when unrealistic doses are given. Further, multiple labs have examined if the plant concentrations of these chemicals are high enough in the field to cause harm to monarchs, and the answer there is also no. Off the top of my head, I can think of the following studies: Prouty et al 2023, Hall et al 2022, Bargar et al 2020, Olaya-Arenas and Kaplan 2019. None of these studies were mentioned at all. Again, an egregious oversight.

We agree that our failure to address the disconnect between our results and lab toxicology studies was an oversight. This issue was also raised in the original submission of the Shirey and Ries (2023) review and that led Ries to undertake a substantial review of the lab toxicology research. This included discussions with the lead author of two of the primary papers on the topic (Niranjana Krishnan). From this exploration, Shirey and Ries arrived at the conclusion that there is, in fact, a disconnect between results in the toxicology literature and observational studies of field effects of pesticides on non-target population sizes. This is especially true of neonicotinoids, which show relatively low toxicity in a laboratory setting for monarchs (Krishnan et al., 2021) and other non-target Lepidoptera (e.g., Basley and Goulson 2018), as we now frame the issue (lines 555-572). There is a substantial literature on that disconnect which suggests that sublethal effects may be driving observed declines in the field and those impacts are difficult to anticipate based on laboratory studies. Given the importance of this topic, we have now added two paragraphs to treat these issues.

There was another result of these analyses that seemed to be purposely overlooked by the authors, but yet would have tremendous implications if this paper were ever to see the light of day. Apparently, there is a statistically significant, positive effect of glyphosate use on butterfly diversity, and at least a positive trend on monarch abundance (Fig. 4A). This result seems to strain credulity.

Reviewer 1 did not point out any deficiencies in our study design; the statement that the results “strain credulity” would have been more useful if they had suggested alternative analysis or identified flaws. We address this issue specifically for monarchs below, but for the general butterfly community, we had stated in our initial submission (with text retained and now on lines 430-433):

*“Here, we note an observed shift in dynamics for species richness starting around 2008 (Fig. 4B, E), a breakpoint that coincides with the proliferation of weed resistance to glyphosate [Box 1; 48] that triggered a switch in the trajectories of glyphosate vs. non-glyphosate herbicide use (Fig. 2J,[51]).”*

We also address the shifting dynamics of pesticide use in Box 1, and the correlations among variables in Table S3. The positive “trend” pointed out for monarchs is based on a coefficient very close to zero and that significantly overlaps zero, a result that we interpret as “zero.”

Not only that, the authors here found NO negative effect of glyphosate use on monarch abundance, even though multiple prior studies (even by these authors) have apparently concluded that glyphosate use in the Midwest was destroying monarch habitat, and leading to their demise.

Ries has never published a paper saying that “glyphosate use in the Midwest was destroying monarch habitat, and leading to their demise.” Instead, she is a coauthor on a major study (“Changes in climate drive recent monarch butterfly dynamics” (Zylstra et al., 2021)) showing that glyphosate in summer accounts for 8% of the estimated variability in the overall summer population size in the Midwest after 2004. An earlier study, focused only in Illinois and Ohio (Saunders et al., 2018) showed a significant impact of glyphosate from 1994-2003, but that this had flattened after 2003. Further, both of these studies included spring climate in Texas as a covariate in the model, which has always been the primary driver of monarch summer population sizes based on multiple studies (Shirey and Ries, 2023). Given that our analysis here does not include Texas climate in March and April and also spans a different temporal extent (1998-2014), a disconnect in results is not entirely surprising. On this topic, in our original submission, we included the statement:

*“Most previous studies have focused on the increased use of glyphosates that occurred with the adoption of herbicide-resistant crops and have caused intense debate within the monarch research community [58]. We found no effect of either glyphosate or non-glyphosate herbicides (Fig. 4G). Our conclusions differed from another study [26], whose analyses encompassed a period before adoption rates of glyphosate-resistant crops reached 75%, a threshold above which glyphosate effects flatten. As our study period includes data after this threshold had been reached, a lack of a statistically significant effect size for glyphosate in our analysis is not inconsistent with previous studies.”*

In keeping to the limited space that we hoped to devote to our monarch discussion, we did not dive into granular details about how to square the disconnect between this and other studies. But we appreciate reviewer 1 pointing out how damaging it could be if our results and conclusions were misconstrued. To address this, we have greatly expanded our attention to this in the manuscript, as noted above.

Yet another important (and credulity-straining) result is that Bt-treated plants are improving the abundance of monarch butterflies, according to/ these analyses. Yet again, here is a result that blows a giant hole into a suite of prior (older) research that suggested that this crop modification would harm monarchs. This research was from the early 2000s, and some of that hoopla later died down because it turns out the monarchs were never exposed to as much contaminated anthers as people thought.

This “hoopla” exploded with a paper in 1999 that suggested that Bt corn could harm monarchs through pollen (Losey et al. 1999). This led to a rapid response from the research community that resulted in a risk assessment instead showing that any impacts of Bt corn on monarchs would be negligible (Sears et al., 2001). This study included two leading researchers on monarch conservation, Karen Oberhauser and John Pleasants, both of whom have been particularly vocal about the negative impacts of glyphosates.

But, this new analysis seems to suggest that Bt-corn is even good for monarch abundance?

Yes, in our discussion, we suggested that it is not counterintuitive that Bt-corn could be good for butterflies, because it is so targeted for species that feed on corn. We added new text in Box 1 that details the history and impact of use of various pesticides. While in our original submission, we did not feel the need to specifically address monarchs since that issue has been basically settled for 20 years, but we now add language to that effect (Box 1).

So with this new paper, the authors want the reader to believe that glyphosate is NOT harming monarch numbers in the Midwest, and if anything, is helping to improve butterfly diversity there! So is Bt-corn! AND, the authors want people to believe that neonicitinoid pesticides are now to blame for all of the woes of butterflies, and especially monarchs (which all lab studies have shown are resistant to neonics). This is why I think the authors need to do some soul-searching here, and decide if they really want to damage their reputation(s) with this paper. By publishing this, the authors will essentially be undermining much of all of this the prior work, which will call into question either this paper, or, the validity of prior analyses. And I haven’t even begun to discuss what would happen if the media caught wind of these new results. The media hoopla over these “new” findings would cause an upheaval and undermine public faith in scientists.

I’m going to assume that this journal will reject the paper, and so these comments are really more for the authors to consider, for gauging whether to resubmit elsewhere. This is probably the harshest review I’ve ever written, but it is because the level of arrogance here is palpable – both for purposely misleading the reader on key issues, and for presenting results that are so at odds with so much recent and established evidence, without even as much as mentioning that evidence. The lack of objectivity here is shocking. Please consider what I said. If I were you, I would table this project. It will be a hot potato if published elsewhere.

Where relevant, we have now accounted for literature on the monarch through expanded discussion in the text.

Reviewer #2: The paper is very well written. The analysis goals of evaluating different drivers of butterfly abundance are interesting and valuable. The dataset is opportunistic but nonetheless well put together (more on that). It covers a useful number of years and locations (more on that). The analysis is well done but could use a few more components for readers to understand possible confounding (more on that). The conclusions are reasonable and are not overblown given the data and analysis methods (more on that). The tables and figures are great. I only have a few thoughts on how to make the paper more convincing (or not).

Thank you for the kind comments and thorough review. We have incorporated your insights into the revised manuscript as noted in the response below.

First, I like the counterfactual analysis as a way of exploring the implications of the model. Given it’s complete dependence on the model, a reader must be convinced that you got the model right if you want them to trust your conclusions. This is a big task in this context, where you have an observational study, two somewhat-vague community-level response variables, a cobbled together dataset, study counties popping in and out of the dataset, and likely loads of correlation among the independent variables. So, here are some thoughts.

First, related to the community metrics. It would be nice to know which species are driving them. Consider creating a plot or table that gives the median abundance per species, like a rank-abundance bar plot. Readers might like to know if the abundance metric is entirely driven by cabbage whites, for example. Or maybe diversity depends entirely on prairie-dependent species. All this will help a reader understand your results better.

In response, we produced a plot of relative abundance by species, now in the Supporting Information as Fig. S2. No single species dominates the total abundance measure. A sentence was added to the Methods section describing the measurement of the butterfly variables (Pg 4, Lines 18-20).

NEW SENTENCE (lines 229-231): “Aggregate counts of individuals identified by species across all county-years show a large number of species detected in the community with no one species dominating the total abundance measures (Fig. S2)”

NEW FIGURE (Supporting Information, Fig. S2):

Fig. S2. Proportion of total abundance across all years and counties by species.

Second, regarding an opportunistic dataset, is there geographic or temporal confounding going on? Can you make something like a map that shows when the different counties enter the dataset? Maybe it is like fig 2D? Or maybe it is a table? With 60 rows and columns summarizing the extent of different data types included?

We present a figure (Fig. S1) showing when counties enter/exit the panel, and a table (Table S2) tallying counties by the number of years they are represented. Eighteen counties are represented only once in the sample, and six more only twice. Note that the original submission misstated the number of counties represented in the panel; the final number of eligible counties was 81. (the Y axis gives county FIPS code)

Table S2

| years in panel | n |
| --- | --- |
| 1 | 18 |
| 2 | 6 |
| 3 | 6 |
| 4 | 6 |
| 5 | 3 |
| 6 | 3 |
| 7 | 1 |
| 8 | 1 |
| 9 | 3 |
| 10 | 4 |
| 11 | 4 |
| 12 | 2 |
| 13 | 2 |
| 14 | 2 |
| 16 | 6 |
| 17 | 12 |

This figure and table are referenced when the county-year unit is defined at the beginning of the Methods section.

NEW SENTENCE (lines 202-203): Counties entered the panel in different years as new monitoring programs were established (Fig. S1).

And, by the way, does having a county with 1 year of data really help for this analysis? Or is it more likely to cause unexpected weirdness?

As we responded in our comments to the editor, our use of all data is appropriate. This is because the GLM analyses are not contingent on time series; rather, they are analyzing the abundances in relation to drivers in each county-year. We checked to see if our results were robust to a restricted panel consisting only of counties contributing data for three or more years. With ~20% fewer counties represented and 5% fewer county-year observations, the standard errors are larger, though the estimates are characteristically similar. These estimates are now discussed in a new paragraph in the Methods section.

NEW SENTENCE (lines 368-371): “We present the full dataset because it permits the most rigorous evaluation due to improved statistical power utilizing all available information, with the reduced dataset showing the sensitivity of our results to sample size (Fig. S4) and the need for ongoing, comprehensive data for all drivers and responses.”

NEW TABLE (Supporting Information, Table S4, Fig. S4):

| Table S4: Generalized linear model (negative binomial) regression summaries. Estimated on panel limited to counties with three or more years of data. | | | |
| --- | --- | --- | --- |
| Term | All species | Distinct species | Danaus plexippus |
| Intercept | -33.1***  (11.6) | 0.0857  (8.65) | -4.2  (8.24) |
| Glyphosate area-treatments | 0.14  (0.287) | 0.485***  (0.141) | 0.0502  (0.167) |
| Non-glyphosate area-treatments | -0.00298  (0.11) | -0.0877  (0.0566) | 0.0471  (0.075) |
| Pyrethroid area-treatments | 0.297  (0.535) | -0.13  (0.27) | -0.171  (0.266) |
| Organophosphate area-treatments | -2.23***  (0.781) | -0.682*  (0.407) | -0.652  (0.421) |
| Neonicotinoid area-treatments | -0.866  (0.556) | -0.187  (0.295) | 0.144  (0.398) |
| Bt seed area-treatments | 0.924*  (0.529) | -0.288  (0.272) | 0.214  (0.319) |
| Forest land cover | -30.5**  (14.4) | 10.4  (8.73) | -17.8*  (10.2) |
| Urban land cover | 19.9**  (8.9) | -5.18  (6.42) | 4.85  (5.86) |
| Cropland land cover | 40.4***  (13.3) | -3.6  (9.89) | 7.44  (9.37) |
| GDD (Early) | 0.00139  (0.00123) | 0.000559  (0.000689) | -0.00126*  (0.000746) |
| GDD (Mid) | -0.0029***  (0.000876) | 0.0004  (0.00045) | -0.000389  (0.000479) |
| GDD (Late) | -0.000144  (0.000993) | 0.000602  (0.000449) | -0.000302  (0.000547) |
| Precipitation (Early) | 0.000184  (0.000622) | -0.0000287  (0.000305) | -0.0000393  (0.000355) |
| Precipitation (Mid) | 0.00108**  (0.000548) | -0.0000428  (0.000291) | -0.00114***  (0.000315) |
| Precipitation (Late) | 0.0000783  (0.000396) | 0.000322  (0.000212) | -0.0000183  (0.000237) |
| Fixed effects | County, Year | County, Year | County, Year |
| Adj. McFadden’s Pseudo R2 | 0.757 | 0.818 | 0.660 |
| Dispersion (theta) | 11.175  (0.665) | 19.05  (1.46) | 4.74  (0.349) |
| AIC | 5285.6 | 5345.4 | 8898.9 |
| BIC | 5677.9 | 5737.7 | 9291.3 |
| N | 607 | 607 | 607 |
| * p < 0.1, ** p < 0.05, *** p < 0.01; Heteroskedasticity-consistent standard errors in parentheses (HC1). | | | |

Figure S4:

Related to this, seems like random effects for counties and years would be better than fixed effects. Maybe using fixed effects is an econometrics thing?

In response to this comment, we estimated the model using the suggested random effects specification and found results, again, largely similar to those in the preferred specification. These results are presented in the Supporting Information (Table S5) and mentioned in the Methods section of the main text. We prefer the fixed effect specification for county and year effects, as this specification does not rely on restrictive assumptions about the distributions of these effects and allows for more flexible relationships between the fixed effects and other coefficients (Wooldridge, 2010).

NEW SENTENCE (lines 371-373): We also estimated the mixed model using random effects rather than fixed effects for county and year using the lme4 package for R[72] (Table S5).

NEW TABLE (Table S5):

| Table S5: Generalized mixed linear model (negative binomial) regression summaries. Full panel. | | | |
| --- | --- | --- | --- |
| Term | All species | Distinct species | Danaus plexippus |
| Intercept | -1.55**  (0.645) | -2.35***  (0.562) | -3.2***  (1.13) |
| Glyphosate area-treatments | 0.0828  (0.146) | 0.264**  (0.124) | 0.096  (0.268) |
| Non-glyphosate area-treatments | 0.0097  (0.0632) | -0.00461  (0.0536) | 0.0445  (0.116) |
| Pyrethroid area-treatments | -0.115  (0.258) | -0.347  (0.226) | 0.29  (0.468) |
| Organophosphate area-treatments | -0.753*  (0.402) | -0.686**  (0.337) | -2.08***  (0.737) |
| Neonicotinoid area-treatments | -0.376*  (0.224) | -0.607***  (0.17) | -0.908*  (0.515) |
| Bt seed area-treatments | 0.228  (0.278) | -0.0621  (0.242) | 1.65***  (0.496) |
| Forest land cover | 2.05**  (0.89) | -0.986  (0.784) | -1.36  (1.49) |
| Urban land cover | 1.4*  (0.715) | -3.02***  (0.631) | 0.687  (1.2) |
| Cropland land cover | 1.27*  (0.676) | -0.949  (0.596) | 1.22  (1.14) |
| GDD (Early) | -0.00071  (0.000604) | 0.000262  (0.000484) | 0.00189  (0.00117) |
| GDD (Mid) | -0.0000325  (0.000382) | 0.000552*  (0.000294) | -0.00267***  (0.000775) |
| GDD (Late) | 0.000547  (0.000377) | 0.000246  (0.000286) | 0.000889  (0.000754) |
| Precipitation (Early) | -0.000273  (0.000335) | 0.000162  (0.000281) | 0.0000628  (0.000609) |
| Precipitation (Mid) | -0.000956***  (0.000278) | 0.0000751  (0.000227) | 0.00136***  (0.00051) |
| Precipitation (Late) | -0.0000305  (0.000229) | 0.000161  (0.000196) | 0.000281  (0.000404) |
| Random effects | County, Year | County, Year | County, Year |
| AIC | 9444 | 5741 | 5647 |
| BIC | 9529 | 5825 | 5732 |
| N | 637 | 637 | 632 |
| * p < 0.1, ** p < 0.05, *** p < 0.01 | | | |

Related to spatiotemporal confounding, you probably know that it’s good practice on these types of large-scale and -extent projects to look for spatial and temporal autocorrelation in model residuals. Think Luke Anselin.

We considered estimating spatial autocorrelation models in the style of Anselin’s work. One feature of both the fixed effect and random effect models is that the county effect, whether directly estimated in the fixed effect model or accounted for with a random effect parameter, accounts for temporally constant unobserved heterogeneity at the county level (Wooldridge 2010). As a result, assuming that the spatial autocorrelation is consistent over time, then the fixed effects will account for any suspected spatial autocorrelation. Swinton found, for example, that incorporating random effects at the household level led to nearly indistinguishable results as a spatial autocorrelation model (Swinton 2001). Considering the significant computational challenges in estimating a model that explicitly accounts for spatial autocorrelation in a panel setting, we chose to prioritize the random effects and restricted panel estimates as evidence of the robustness of our results to alternative assumptions.

Third, I imagine there is plenty of correlation among your independent variables. Maybe it is not enough to cause computational problems (extreme multicollinearity). But it is probably enough to cause interpretation challenges. In this case, it is helpful to run univariate correlations or regression models and note the model coefficients. It sure is convenient, and ultimately more convincing, when the univariate effect estimates are similar to the multivariate effect estimates. When that is not the case, due to independent variable correlations, you have to be extra careful in interpreting model results, right?

We constructed a matrix showing Pearson’s correlation coefficient for all variables, along with the Variance Inflation Factors (VIFs) for each. While there are strong correlations between some variables, their variance inflation factors are all fairly reasonable, perhaps with the exception of the land cover variables, which are included as controls and not strongly correlated with the pest control variables. We discuss this figure in text in the Methods section.

NEW SENTENCE (lines 358-364): “In support of our GLM estimates, we present a plot of pairwise correlations and Variance Inflation Factors (VIFs) for each explanatory variable (Fig. S3). Strong correlation between explanatory variables can induce multicollinearity in GLM estimates that results in inflated variances in coefficient estimates but does not affect the statistical consistency of the estimator[71]. VIFs were fairly low for the pesticide use variables, despite fairly high pairwise correlations between some of these variables.”

NEW FIGURE (Supporting Information, Figure S3):

**Figure S3. Pairwise Pearson’s correlation coefficients and Variance Inflation Factors for explanatory variables in the full panel.**

We estimated each of the three models six additional times, once for each pesticide use covariate where the other five covariates were excluded. The coefficient estimates on the relevant pesticide covariate for each of the eighteen supplemental models are presented below. While there is little agreement in between the univariate coefficient estimates and the preferred specification with the full suite of pesticide variables, this is expected given the correlations and substitution patterns between each pest control method. This additional analysis highlights the risk of focusing on a single pesticide at once. Additional discussion on the expected differences in coefficient estimates expected when relevant, correlated variables are omitted is available in Wooldridge, 2010, Chapter 3 for the linear case. A similar argument extends to GLM estimators.

The table below shows the results of these estimates. This table is included in the supplementary material as Table S6. Each row shows the coefficient estimate for a model fitted with just the single pest control variable indicated included, alongside the weather and land cover controls and the county and year fixed effects.

To the reviewer’s point about interpretation challenges, the challenge is in interpreting individual coefficient estimates while considering all of the other variables as “being held equal”, which we know is not the case due to substitution patterns between chemicals. The counterfactual analysis is intended to assist with this challenge by showing the implications of the estimated coefficients under patterns observed in the real world (the substitution patterns between chemicals based on farmers’ actual choices). These results account for how the several coefficients and observed patterns in the data interact when used to predict abundances under alternative conditions, rather than looking at partial effects in isolation.

| Total Abundance | |  |  |  |
| --- | --- | --- | --- | --- |
|  | Estimate | Std. Error | z value | Pr(>\|z\|) |
| Glyphosate | 0.146 | 0.145 | 1.013 | 0.311 |
| Non glyphosate | -0.026 | 0.067 | -0.392 | 0.695 |
| Pyrethroids | -0.378 | 0.233 | -1.622 | 0.105 |
| Organophosphates | -0.796 | 0.343 | -2.320 | 0.020* |
| Neonicotinoids | 0.406 | 0.329 | 1.237 | 0.216 |
| Bt traited seed | 0.191 | 0.282 | 0.679 | 0.497 |
|  |  |  |  |  |
| Species Richness | |  |  |  |
|  | Estimate | Std. Error | z value | Pr(>\|z\|) |
| Glyphosate | 0.511 | 0.131 | 3.900 | 0.00009613*** |
| Non glyphosate | -0.162 | 0.053 | -3.052 | 0.0022715** |
| Pyrethroids | -0.568 | 0.231 | -2.463 | 0.0137904* |
| Organophosphates | -1.030 | 0.337 | -3.055 | 0.0022537** |
| Neonicotinoids | 0.256 | 0.261 | 0.980 | 0.327145 |
| Bt traited seed | 0.047 | 0.257 | 0.181 | 0.856477 |
|  |  |  |  |  |
| Monarch | |  |  |  |
|  | Estimate | Std. Error | z value | Pr(>\|z\|) |
| Glyphosate | 0.295 | 0.255 | 1.156 | 0.24755 |
| Non glyphosate | -0.095 | 0.107 | -0.892 | 0.372277 |
| Pyrethroids | -0.201 | 0.480 | -0.417 | 0.67641 |
| Organophosphates | -1.590 | 0.623 | -2.555 | 0.0106212 * |
| Neonicotinoids | 0.076 | 0.493 | 0.154 | 0.877357 |
| Bt traited seed | 0.783 | 0.465 | 1.682 | 0.0924891 . |

Why all this annoying model scrutiny? Because if your model is not a slam dunk then any counterfactual analysis, and subsequent conclusions, and subsequent someday ag or environmental policies, and subsequent economic impacts on real people, are not well founded. You could probably publish this paper without any of this additional junk. But if you do the extra work and still come to the same conclusions, then your conclusions will be that much more powerful and valuable. From a less lofty perspective, the monarch mafia is going to have a hard time believing that monarch decline is more about seed coat treatment than glyphosate application or loss of hedgerows due to corn ethanol. So, you’ll need to convince them your results are not confounded by correlations between independent variables.

We wholeheartedly agree and appreciate the thoughtful comments. These thoughtful criticisms provided a clear roadmap for robustness checks that confirm our findings and enhance the credibility of our conclusions. Thank you for the kind hearted remarks and guidance.

Reviewer #3: I enjoyed reading this manuscript; it is rigorous and important piece of work. In particular, the scientific approach of contrasting alternative predictors for a problematic species’ (in this case, declining) population abundance is critical to get past simple correlations. Much past work on the focal species examined here has not taken this approach and provided on parts of the story, some of which suffered from correlations. Finally, the authors present a complete and balanced picture. I particularly appreciated the distinction of insecticides vs. herbicides in this study. Finally, the 17 year scope of the work, the use of multiple abundance databases, and the diversity of butterflies covered makes the study of exceptional importance.

Although I am not familiar with the specifics of counterfactual analysis, I did find the data summary, statistics, and reporting convincing.

There are clear policy implications of this work.

Figures are excellent.

Thank you for these kind remarks. It is always helpful to receive highlights of where our work is strongest.

Reviewer #4: Review of “Neonicotinoids, more than herbicides, land use, and climate, drive recent butterfly declines in the American Midwest”
This is an ambitious study of existing data, attempting to demonstrate a spatio-temporal correlation of butterfly richness, numbers, and specifically monarch numbers with various factors, including different aspects of herbicide and pesticide use. The authors draw a strong conclusion about the negative effects of neonicotinoids based on the analysis. Given the inevitable controversy that monarch research, especially insecticide-related, generates, there must be more clarity in the methods and discussion of uncertainties for this to be an effective contribution.

Extracting trend data from butterfly monitoring programs can be tricky, although strong signals usually come through the noise. Time series have many factors correlated with time itself, and the combination of counterfactual analysis and the negative binomial GLM attempts to disentangle the factors.

But, the following aspects of the analysis need clarification:
A key figure would be the raw time series of species richness, overall abundance, and monarch counts with the same format as in Figure 2 – a mean/median line with the cloud of county level data.

We created the requested figure and included it in a revised Figure 2 in panels A-C. The revised figure is copied below for reference.

REVISED FIGURE (Fig. 2):

**Figure 2. Data summaries for key variables.** (A-C) shows land cover by county as percent of total land area in 2014 and (D) shows the number of years each county contributed butterfly population surveys; bold borders indicate USDA Crop Reporting District (CRD) boundaries while hatching shows counties not included in analysis due to lack of butterfly survey data availability. (E-G) shows the distributions of annual county-level species richness, total abundance, and monarch abundance measures; each line represents an individual county and the bold line represents the median value in each year. (H-J) shows area-treatments, the average number of times a pesticide or pest control treatment is applied to an acre of soybean or corn cropland, for each pest control technology group; each line represents a CRD and the bold line represents the median value in each year.

The period 1998-2014 had data from 78 counties (SI table). The abstract says 60 counties. Which 60? (in the S.I Table). Is it the counties with at least 2 years? Is that how many faded lines there are in each graph in Figures 2 and 3? It is not clear how the interrupted time series of county-level counts were handled – 28 counties had only 1-4 years of data, while 13 had complete time series. The inclusion of the “Year” and “County” co-variables in the GLM (maybe?) addressed this - i.e., factors well beyond the study region, like the 2011 drought in Texas and winter storms in Mexico in the “Year” factor, and the sampling incompleteness in the "County" factor, but much more explanation needs to be made, especially the counties that had 1-4 (or 2-4) years data. Could be in the SI.

This comment is similar to a comment by Reviewer 2. We repurpose the response above here for convenience.

To provide more transparency into the unbalanced nature of the panel, we present a new figure (Fig. S1) showing when counties enter/exit the panel, and a new table tallying counties by the number of years they are represented (Table S1). Eighteen counties are represented only once in the sample, and six more only twice. Note that the original submission misstated the number of counties represented in the panel; the final number of eligible counties was 81. This figure and table are referenced when the county-year unit is defined at the beginning of the Methods section.

NEW SENTENCE (lines 202-203): “Counties entered the panel in different years as new monitoring programs were established (Fig. S1).”

NEW FIGURE (Supporting Information, Fig. S1):

**Fig. S1. Years each county contributes to the panel.** Bold dots indicate first year data is contributed. The vertical axis shows the county’s FIPS code.

NEW TABLE (Supporting Information, Table S1):

Table S1: Tally of counties by number of years in the panel

| **Years in panel** | **N, counties** |
| --- | --- |
| 1 | 18 |
| 2 | 6 |
| 3 | 6 |
| 4 | 6 |
| 5 | 3 |
| 6 | 3 |
| 7 | 1 |
| 8 | 1 |
| 9 | 3 |
| 10 | 4 |
| 11 | 4 |
| 12 | 2 |
| 13 | 2 |
| 14 | 2 |
| 16 | 6 |
| 17 | 12 |

We checked to see if our results were robust to a restricted panel consisting only of counties contributing data for three or more years. With ~20% fewer counties represented and 5% fewer county-year observations, the standard errors are larger, though the estimates are characteristically similar. These estimates are now discussed in a new paragraph in the Methods section.

NEW SENTENCE (lines 368-371): “We present the full dataset because it permits the most rigorous evaluation due to improved statistical power utilizing all available information, with the reduced dataset showing the sensitivity of our results to sample size (Fig. S4) and the need for ongoing, comprehensive data for all drivers and responses.”

NEW TABLE and Figure (Supporting Information, Table S4, Fig. S4)

In trying to interpret the results, is it correct say that the leverage of each factor is dependent on the spatial variability among counties in, say, insecticide treatments, and the sharp increase in neonicotinoids from 2003-2009?

Yes this is it exactly. The differences in adoption rates between treatments across counties, and the explicit inclusion of pesticides that were adopted or disadopted at similar but distinct rates due to substitution or complementarity effects, allows for the identification of associations between the amount of each treatment on the landscape and the butterfly variables that control for these substitution and complementarity effects. Because farmers do not adopt pesticide treatments in isolation, rather they substitute out existing treatments when more effective alternatives become available, examining a pesticide treatment in isolation risks omitted variable bias. This is now addressed more explicitly in the revised manuscript in the new Box 1.

A very recent paper (not available when this MS was written) has some methods for splitting the time series into periods corresponding to changing factors like herbicide use, since the elimination of within field milkweeds was nearly complete by 2006. The discussion on p.14 of glyphosate hints at this break, but more explicit consideration
(Pleasants, J., Thogmartin, W.E., Oberhauser, K.S., Taylor, O.R. and Stenoien, C., 2023. A comparison of summer, fall and winter estimates of monarch population size before and after milkweed eradication from crop fields in North America. Insect Conservation and Diversity.)

We thank the reviewer for raising our awareness of this paper. We did not add a new analysis in this paper, but we will consider it in future analyses.

The inclusion of the annual weather data by county is an important control. But the lack of post 2014 pesticide data prohibits consideration of the more recent hottest years (as mentioned on p. 14). Given that neonicotinoids leveled off after 2009, could a similar counterfactual analysis of climate impacts since 2014 reveal more about the weather impacts?

Without complete pesticide data through the present, extending the model estimates through the present would not be feasible. Even though neonicotinoid use appears to steady off in 2009, there is no way to be sure that adoption levels did not dip or spike further in the past decade. We feel that assuming that they remain steady without data would not be defensible in this context.

And some of the other significant results from the GLM deserve more discussion (like species richness-glyphosate positive)

We have added detailed discussion of other variables, especially in our original GLM analysis, and its implications for counterfactual analyses that consider groups of insecticides and pesticides in three paragraphs from Lines 473-510.

The proportion of butterflies in Mexico from this region should be reported as a range, rather than a single value (P.13). It does change from year to year, but there is no doubt that this region serves as a major source of migratory monarchs.

Reviewer 1 also indicated some concerns about our treatment of this. We edited our reference to Flockhart, et al. 2017 to indicate the Midwest is the major source of eastern monarchs.

The published literature on experimentally determined lethal and sublethal effects of neonicotinoids is confusing; some studies show minimal or no effects at field relevant concentrations, other show important mortality and sublethal effects. While neonicotinoids (and other insecticides) undoubtedly kill their targets, it is important to acknowledge the disparate results.

We have now included a paragraph on these discrepancies for Monarchs (lines 555-572). We also discuss how butterflies under field conditions are confronted with ongoing, sublethal effects that may in sum prove lethal.

**Literature Cited**

Basley, K., & Goulson, D. (2018). Effects of field-relevant concentrations of clothianidin on larval development of the butterfly Polyommatus icarus (Lepidoptera, Lycaenidae). *Environmental science & technology*, 52(7), 3990-3996.

Boyle, J.H., Strickler, S., Twyford, A., Ricono, A., Powell, A., Zhang, J., Xu, H., Dalgleish, H.J., Jander, G., Agrawal, A.A. and Puzey, J.R., 2022. Temporal matches and mismatches between monarch butterfly and milkweed population changes over the past 12,000 years. *bioRxiv*, pp.2022-02.

Crossley, M. S., Smith, O. M., Berry, L. L., Phillips‐Cosio, R., Glassberg, J., Holman, K. M., ... & Snyder, W. E. (2021). Recent climate change is creating hotspots of butterfly increase and decline across North America. *Global Change Biology*, 27(12), 2702-2714.

Flockhart, D. T., Brower, L. P., Ramirez, M. I., Hobson, K. A., Wassenaar, L. I., Altizer, S., & Norris, D. R. (2017). Regional climate on the breeding grounds predicts variation in the natal origin of monarch butterflies overwintering in Mexico over 38 years. *Global change biology*, 23(7), 2565-2576.

Inamine, H., Ellner, S. P., Springer, J. P., & Agrawal, A. A. (2016). Linking the continental migratory cycle of the monarch butterfly to understand its population decline. *Oikos*, *125*(8), 1081-1091.

Krishnan, N., Zhang, Y., Aust, M. E., Hellmich, R. L., Coats, J. R., & Bradbury, S. P. (2021). Monarch Butterfly (Danaus plexippus) Life‐Stage Risks from Foliar and Seed‐Treatment Insecticides. *Environmental Toxicology and Chemistry*, *40*(6), 1761-1777.

Losey, J. E., Rayor, L. S., & Carter, M. E. (1999). Transgenic pollen harms monarch larvae. Nature, 399(6733), 214-214.

Normile, D. (2023). Monarch butterfly is not endangered, new review finds. *Science (New York, NY)*, *382*(6666), 17-1.

Ries, L., Oberhauser, K., Taron, D., Battin, J., Rendon-Salinas, E., Altizer, S., & Nail, K. (2015). Connecting eastern monarch population dynamics across their migratory cycle. *Monarchs in a changing world: Biology and conservation of an iconic butterfly*, 268-281.

Saunders, S. P., Ries, L., Oberhauser, K. S., Thogmartin, W. E., & Zipkin, E. F. (2018). Local and cross‐seasonal associations of climate and land use with abundance of monarch butterflies Danaus plexippus. *Ecography*, *41*(2), 278-290.

Sears, M. K., Hellmich, R. L., Stanley-Horn, D. E., Oberhauser, K. S., Pleasants, J. M., Mattila, H. R., ... & Dively, G. P. (2001). Impact of Bt corn pollen on monarch butterfly populations: a risk assessment. *Proceedings of the National Academy of Sciences*, 98(21), 11937-11942.

Shirey, V., & Ries, L. (2023). Population dynamics and drivers of the eastern monarch (Danaus plexippus) across its full annual cycle: a cross-scale synthesis of a model migratory species. *Current Opinion in Insect Science*, 101132.

Swinton, Scott M. “Capturing Household-Level Spatial Influence in Agricultural Management Using Random Effects Regression.” *Agricultural Economics* 27, no. 3 (2002): 371–81. <https://doi.org/10.1111/j.1574-0862.2002.tb00126.x>.

Taron, D., & Ries, L. (2015). Butterfly monitoring for conservation. In *Butterfly conservation in North America: efforts to help save our charismatic microfauna* (pp. 35-57). Dordrecht: Springer Netherlands.

Thogmartin, W. E., Szymanski, J. A., & Weiser, E. L. (2020). Evidence for a growing population of eastern migratory monarch butterflies is currently insufficient. *Frontiers in Ecology and Evolution*, 8, 43.

Wooldridge JM. Econometric Analysis of Cross Section and Panel Data. MIT Press; 2010.

Van Klink, R., Bowler, D. E., Gongalsky, K. B., Swengel, A. B., Gentile, A., & Chase, J. M. (2020). Meta-analysis reveals declines in terrestrial but increases in freshwater insect abundances. *Science*, *368*(6489), 417-420.

Zylstra, E.R., Ries, L., Neupane, N., Saunders, S.P., Ramírez, M.I., Rendón-Salinas, E., Oberhauser, K.S., Farr, M.T. and Zipkin, E.F., 2021. Changes in climate drive recent monarch butterfly dynamics. *Nature Ecology & Evolution*, *5*(10), pp.1441-1452.
